# A Bibliometric Analysis of the Association Between Compassion Fatigue and Psychological Resilience From 2008 to 2021

**DOI:** 10.3389/fpsyg.2022.890327

**Published:** 2022-06-22

**Authors:** Li-Juan Yi, Yi Liu, Ling Tang, Liang Cheng, Guo-Hao Wang, Su-Wen Hu, Xiao-Ling Liu, Xu Tian, Maria F. Jiménez-Herrera

**Affiliations:** ^1^Department of Nursing, Hunan Traditional Chinese Medical College, Zhuzhou, China; ^2^Department of Nursing, Universitat Rovira I Virgili, Tarragona, Spain; ^3^Department of Physical Education, Hunan Traditional Chinese Medical College, Zhuzhou, China; ^4^Chongqing University Cancer Hospital, Chongqing, China; ^5^Department of Management Engineering, Tangshan Industrial Vocational & Technical College, Tangshan, China; ^6^Chongqing Key Laboratory for Intelligent Oncology in Breast Cancer (iCQBC), Department of Ultrasound, Chongqing University Cancer Hospital, Chongqing, China

**Keywords:** compassion fatigue, physiological resilience, Web of Science, bibliometric analysis, COVID-19

## Abstract

**Aims:**

A negative association between the lower level of psychological resilience (PR) and increased risk of compassion fatigue (CF) and higher Coronavirus disease 2019 (COVID-19) stress has been revealed. However, bibliometric studies have not been performed to comprehensively investigate this topic. This study aimed to identify the status and trends in the CF and PR field from 2008 to 2021 and during the COVID-19 pandemic.

**Methods:**

We identified relevant literature from the Web of Science Core Collection^®^ database using “resilience” and “compassion fatigue” on September 30, 2021. All search results were exported in plain text format for collaboration network analysis, reference-based co-citation analysis, analysis of journals, and keywords-based co-occurrence analysis, which were performed using Citespace^®^ 5.8.R1.

**Results:**

A total of 388 publications were identified finally, and there has been an increasing trend in the annual number of publications with light fluctuations. The analysis of journals and keywords indicated that nurses and social workers are the main research targets, and their mental problems are the main research topics. The turnover intention of health care providers has been a research focus, particularly during the COVID-19.

**Conclusion:**

The results of the present study help us understand the status of the CF and PR field and its recent developments.

## Highlights

- The number of publications focused on compassion fatigue (CF) and psychological resilience (PR) increased rapidly, particularly during the COVID-19 pandemic.
- CF and PR are becoming a hot research topic, especially the psychological problems of nurses and social workers.
- The turnover intention of healthcare providers has been focused on during the COVID-19.
- Research on CF and PR is at the initial stage, and more studies should be performed to investigate the effective strategy for cultivating PR.

## Introduction

Compassion fatigue (CF) refers to emotional, physical, and psychological exhaustion distress resulting from repeated or prolonged expression of compassion or empathy, which may occur in individuals working in caregiving professions (Stamm, 1995, 2010; Hernández et al., 2007; Skovholt and Trottermathison, 2011). Currently, different terms, such as vicarious stress, secondary traumatic stress, burnout, and compassion stress, have been used interchangeably with the term CF (Joinson, 1992; Figley, 2002; Gentry, 2002); however, it can be divided into two parts including burnout (BO) and secondary traumatic stress (STS) (Gerami Nejad et al., 2019). A systematic review involving 28,509 nurses from 11 countries revealed a pooled mean BO of 26.64 and STS of 25.24, respectively, suggesting that nurses suffered from significant CF. Meanwhile, the authors also uncovered levels of CF increased gradually from 2010 to 2019, reaching a peak in 2019 (Xie et al., 2021). Besides, a cross-sectional survey study by Khalaila (2021) stated that CF is prevalent among family caregivers and showed a positive correlation between caregiver burden and CF. Long-term effects of CF aggravated the development of job stress and BO and then led to a low level of work engagement, posing challenges for staff retention and influencing patient safety (Craig and Sprang, 2009; Maiden et al., 2011; Thomas, 2013; Mason et al., 2014; Khamisa et al., 2015; Kim et al., 2017; Zhang et al., 2018; Wood et al., 2019; Allday et al., 2020).

Coronavirus disease 2019 (COVID-19), an unprecedented global health crisis (Har and Snbbc, 2020), has infected over 243 million people and caused 5 million deaths as of 25 October 2021 (Johns Hopkins University, 2021). Evidence showed that service providers from different fields are highly susceptible to psychological problems under the high-pressure and high-risk situations, which was also demonstrated under COVID-19 conditions (Shih et al., 2009; Kang et al., 2015; Alharbi et al., 2020b; Louise Duncan, 2020; Ran et al., 2020; Tominaga et al., 2020; Tang et al., 2021). As one of those suffering the most, health professionals are reporting increases in CF, or an inability to care and provide comfort for patients owing to the overwhelming nature of the task at hand (19). According to a newly published online survey, about two in five (*n* = 306) healthcare professionals experienced medium to high CF and BO, especially those who are directly caring for patients with COVID-19 (Ruiz-Fernandez et al., 2021). Meanwhile, other essential professionals, such as psychological hotline counselors, marriage and family therapists, and education professionals, have also been found to encounter more intense BO and CF than ever during the COVID-19 pandemic (Clark et al., 2021; Pérez-Chacón et al., 2021; Zhang et al., 2021a). Consequently, action to prevent or reduce CF cannot be postponed with the ongoing pandemic.

Despite a rich literature on CF in service practitioners, however, very limited information is available on effective initiatives to alleviate it (Cao et al., 2021b; Khalaila, 2021; Salur and Yildirim, 2021; White et al., 2021; Xie et al., 2021; Yeung et al., 2021; Yi et al., 2021). One potentially promising approach is to strengthen psychological resilience (PR) (Zhang et al., 2021b). PR is a positive adaptation process under adversity, stressors, and traumatic events (Campbell-Sills and Stein, 2010), which has been proven to be a protective factor against the development of CF and COVID-19 stress (Burnett and Wahl, 2015; Li et al., 2021; Iimura, 2022). Resilient caregivers would be more likely to cope more effectively with stress and trauma (Alharbi et al., 2020a; Allday et al., 2020; Flanders et al., 2020; Gonzalez-Mendez et al., 2020; Grauerholz et al., 2020) and are also apt to be more optimistic and flexible (New et al., 2009). PR not only decreases anxiety and depression but also fosters job satisfaction and staff retention (Jo et al., 2020; Cao and Chen, 2021a; Labrague and de Los Santos, 2021; Merlo et al., 2021; Muomah et al., 2021). However, the efficacy of resilience promotion intervention on CF has only been confirmed by very few low-quality trials with a small sample of 11, thus, the CF and PR fields are still a research topic, particularly in the context of the pandemic (Sood et al., 2011; Mealer et al., 2014; Gerhart et al., 2016; Michael et al., 2019; Brassington and Lomas, 2020).

Bibliometrics is a scientific approach to analyzing knowledge carriers with mathematical and statistical methods, and it can reveal the trend of a specific research topic (Nicolaisen, 2010; Vaneck and Waltman, 2010). As a popular information visualization software developed in 2004 (Chen, 2006; Chen et al., 2019), CiteSpace benefits from visually showing now the network patterns of a research topic, such as identifying rapidly growing subject areas and discovering and tracking citation hotspots (Chaomei et al., 2012). Many studies have been carried out on CF and PR over the last decade. However, few research studies have outlined this research domain from the perspective of bibliometric analysis.

In the present study, we aimed to identify annual publications and most representative disciplines and journals in the CF and PR field and to unfold which research frontiers could contribute to collaborations between countries, institutions, and authors. Furthermore, we attempted to describe the intellectual landscape of studies and predict future development trending in this field. Finally, we also presented the above content in the view of the COVID-19 pandemic, aiming to analyze and predict trends and hot spots in this special period.

## Materials and Methods

### Design

This was a descriptive bibliometric analysis and science mappings, which was performed to identify and analyze literature on CF and PR. Meanwhile, we performed a separate analysis to determine the impact of the COVID-19 pandemic on research trends. Certainly, we also made a comparison between the overall findings and the COVID-19 pandemic.

### Sample

We retrieved targeted literature from the Web of Science (WOS) Core Collection^®^ from Clarivate Analytics because it allowed us to perform a precise and specific analysis of publications, authors, citations, and keywords (Aggarwal et al., 2016; Ma et al., 2022).

### Data Collection

We performed a systematic search in Web of Science Core Collection^®^, which includes Science Citation Index expanded (SCI-EXPANDED), Social Science Citation Index (SSCI), Arts and Humanities Citation Index (A&HCI), Conference Proceedings Citation Index-Social Sciences & Humanities (CPCI-SSH), and Emerging Science Citation Index (ESCI), to identify relevant literature labeled as article or review. The search was limited from its inception until September 30, 2021. The following search strategy was used to conduct a thorough search: TS (“compassion fatigue” or “vicarious trauma” or “vicarious traumas” or “secondary traumatization” or “secondary trauma” or “secondary traumas” or “secondary traumatization” or “vicarious traumatization”) and TS = (“resilience*”). We did not impose any restrictions on our literature search, such as countries, categories, and language. We show the search strategy in Supplementary Appendix 1.

### Ethical Consideration

No institutional ethical approval and patient’s informed consent were necessary because the present study did not recruit animal or human subjects.

### Data Analysis

Data analysis was performed by using CiteSpace 5.8.R1 and Microsoft^®^ Excel 2013 (Pan et al., 2018). Specifically speaking, Microsoft^®^ Excel 2013 was used to visually analyze the trends of annual publications. However, CiteSpace was applied to produce visualized maps for analyzing the distribution of research fields, cooperation relationships of authors, institutions, and countries, clusters of co-citing reference publications, and the intellectual landscape of studies about CF and PR by time. Additionally, co-occurring keywords were also analyzed by Citespace to detect research trends and frontiers. We defined the following parameters for CiteSpace: time-slicing was from January 2008 to October 2021 and from December 2019 to October 2021, respectively, with years per slice (slice length = 1). All options in the term source were selected, a node type was selected at a time according to specific conditions, “top 50 levels” as a threshold that are cited or most frequent in the corresponding time slice. For a visualization knowledge figure, node and link were the essential elements. A node represented an element, such as author, keyword, or institution, and the size of a node was proportional to the frequency of appearance or citation, and the color of the node indicated the year. Besides, each node is described with a series of tree rings across the series of time slices. The size of the concentric circles represents the number of publications. Also, the circles of different colors signified the year 2008 to 2021 or 2019 to 2021 from the inside to the outside of the nodes. The link between two nodes represented cooperation or co-occurrence or co-citation relationship. The purple ring represented the betweenness centrality (BC) of literature. If a knowledge map appeared with nodes with high BC value, these nodes were believed to bridge different stages of the development of a scientific field (Chen et al., 2009). Red circles indicate the time slices in which citation bursts or abrupt increases of citations are detected (Kleinberg, 2002).

## Results and Discussion

### Annual Trends in Publications and Citations

**FIGURE 1:**
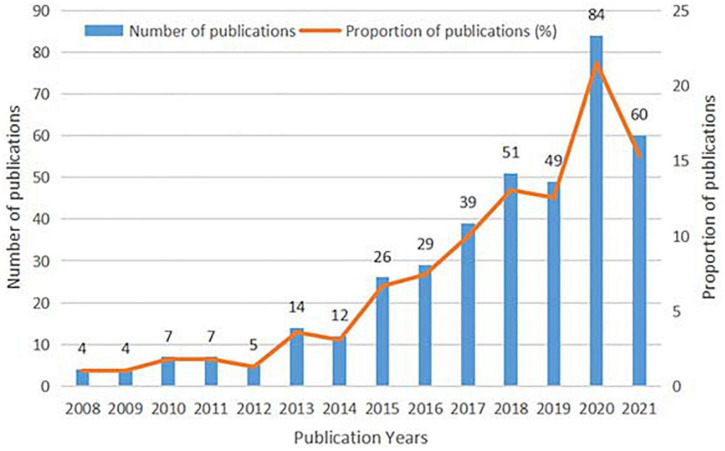
The distribution of the bibliographic records per year.

Overall, 391 records published between 2008 and 2021 were analyzed. Among 391 identified studies, a total of 8 categories were determined, including narrative review (*n* = 64), animal study (*n* = 1), systematic review (*n* = 13), perspective (*n* = 23), interventional study (*n* = 40; self-control trial: *n* = 29, clinical control trial: *n* = 8; randomized controlled trial: *n* = 3), study protocol (*n* = 1), survey study (*n* = 167), and qualitative study (*n* = 82). As shown in Figure 1, research on the association between CF and PR can be roughly separated into two periods. In the first stage from 2008 to 2012, the first article on target research dates to 2008, and we saw fluctuations in the volume of publications at a level of <10. In the second stage from 2013 to 2021, the number of publications increased rapidly, despite two downward trends detected in 2014 and 2019. This indicated that CF and PR have been gradually becoming hot research topics. Besides, to the search date, papers analyzed in this study were cited 5,170 times totally, and the average number of citations was 13.22.

During the COVID-19 pandemic, the number of publications increased rapidly in the field of CF and PR, accounting for 45.01% of 391 publications. Among them, 22 documents are reviews and 154 documents are original articles. The change revealed that CF, as a kind of negative emotional state, has aroused much attention, and more studies have been conducted to explore the impact of a pandemic on the level of CF and PR.

### Category Analysis

**FIGURE 2:**
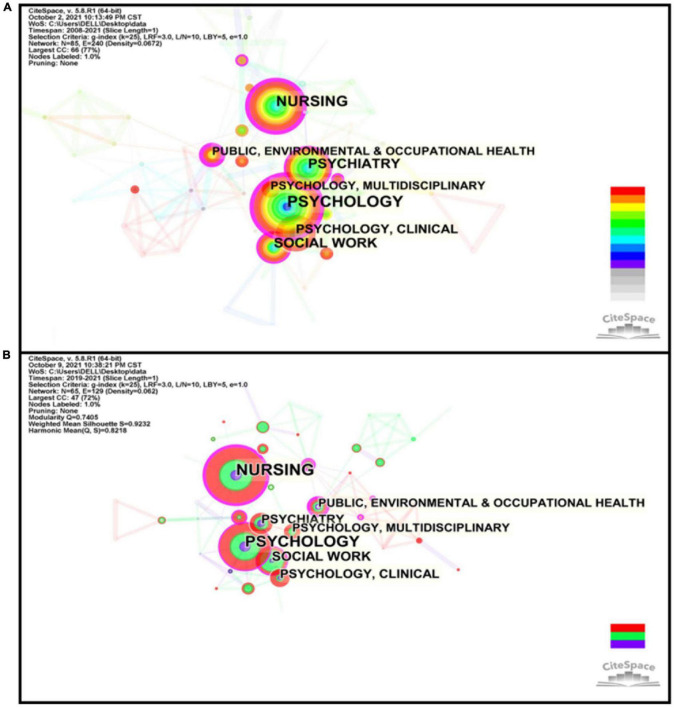
The co-occurrence network of subject categories in the field of compassion fatigue (CF) and psychological resilience (PR) during 2008–2021 **(A)** and the COVID-19 pandemic **(B)**. A circle indicates a subject category. The various colors of nodes indicate the different years, and the size of a circle was weighted by the amount of literature on the category. The link between two nodes represents the interdisciplinary interaction of the literature, and the thickness of the lines was weighted by the relevance between different areas of research. The purple rims of circles indicate high betweenness centralities.

**FIGURE 3:**
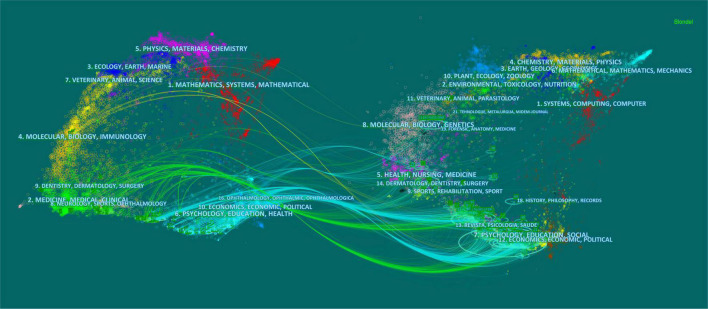
The dual-map overlay of journals. Citing journals are on the left and cited journals are on the right, and the colored paths between them suggested the cited relationships.

The top 10 frequency and centrality of subject categories related to CF and PR indexed by the Web of Science Core Collection^®^ were illustrated by a visualizing calculation result using CiteSpace (see Figure 2A and Supplementary Table 1A). As shown in Figure 2A, 85 nodes and 240 links constituted a network of such subject categories. Among them, the most common category is psychology (93 articles), followed by nursing (77 articles), psychiatry (58 articles), social work (48 articles), and Psychology and clinical (45 articles). The first in the centrality was public, environmental, and occupational health (36). Besides, we also had a dual map-based portfolio analysis of 391 articles. Also, the result showed that citing journals (on the left-hand side of the base map) is associated with topics including medicine, psychology, health, education, and clinical and immunology. The majority clusters of the cited journal (on the right-hand side of the base map) were directed to disciplinary areas, such as medicine, psychology, health, education, nursing, and social (shown in Figure 3).

During the COVID-19 pandemic, the top 10 subject categories with the greatest number of publications were essentially the same (see Figure 2B and Supplementary Table 1B). It is worth noting that education and education research (11 publications) entered the top 10. In this special area, a total of five studies focused on the topic of self-care practice and resilience-based interventions. Self-care is the practice of behaviors that promote well-being, counter work-related stress, and develop resilience (Griffiths et al., 2019; Allison et al., 2020; Keesler and Troxel, 2020; Pandya, 2020; Seo et al., 2021). More research is needed to construct optimal methods to foster resilience and explore better interventions to build up self-care strategies. The top 5 research areas for centrality remained the same (see Figure 2B and Supplementary Table 1B). These reflected that these research categories are very important in bridging cooperative relationships in the CF and PR fields.

### Overview of the Research Co-operation

Visualized knowledge mapping provides information on influential research teams and potential collaborators and helps researchers establish collaborative relationships (Liang et al., 2017).

### Distribution of Countries and Institutions

**FIGURE 4:**
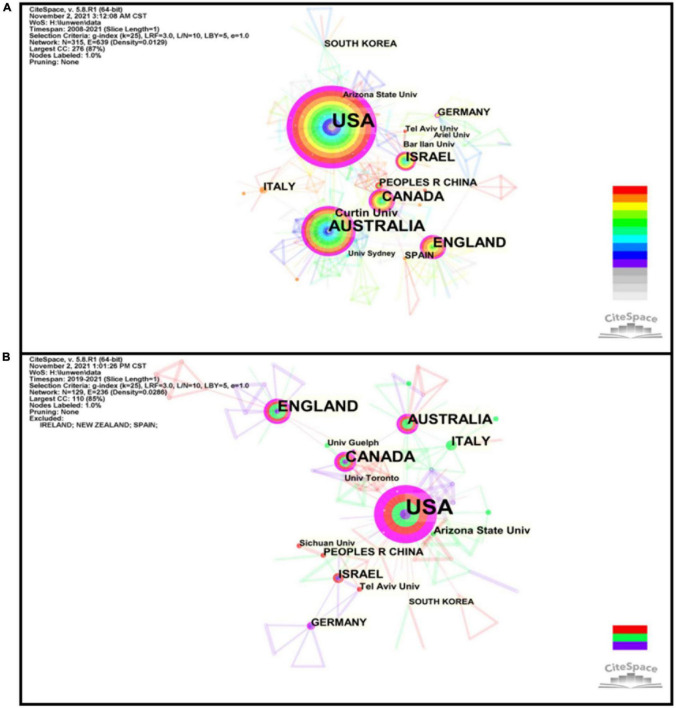
Institutions-countries hybrid network of publications in the field of CF and PR during 2008–2021 **(A)** and the COVID-19 pandemic **(B)**. The individual circle indicates one country or institution, and the size of the circle was weighted by the number of articles published by countries or institutions. The link between two nodes represents partnership, and its thickness was weighted by the frequency of collaborations. The purple rims of circles indicate high betweenness centralities.

According to an analysis of the country distribution of these publications, the top 4 countries for centrality were the United States (0.41), Canada (0.36), England (0.22), and Australia (0.17), all of which also entered the top 4 countries with the largest number of articles (see Supplementary Table 2A). These results showed that contributions from the United States, Canada, England, and Australia cannot be ignored in the field of CF and PR. It is worth noting that the United States was not only the most prolific country (153 articles) but also the highest centrality country, revealing United States is highly outstanding for researching CF and PR, both quantitatively and qualitatively. Indeed, it was these countries that were among the first to call for a focus on CF and PR. Supplementary Table 3A shows the top 10 institutions by the number of articles published. Most academic institutions, which are active in the field of CF and PR have been formed in these top four countries. Furthermore, the remaining three institutions are in Israel, reflecting that we cannot neglect contributions from Israel in this field. The distribution of countries and institution map was generated, with 317 nodes and 624 links composed of the merged network (see Figure 4A). We can see that links between the nodes representing institutions or countries are not much, which means that the interaction and the corporation between institutions or countries in the field of CF and PR are relatively poor.

During the COVID-19 pandemic, as shown in Supplementary Table 2B, the United States (71 articles), Canada (19 articles), and England (19 articles) were still pioneers in this field. Also, the majority of the most productive institutions were located in these developed countries (Supplementary Table 3B). It is also worth mentioning that Sichuan University, a Chinese institution, entered the top 10. This could be because the outbreak of COVID-2019 first emerged in Wuhan, China, and Chinese scholars are taking an interest in this field. Four studies (Cao and Chen, 2021a,b; Cao et al., 2021a,b) from Sichuan University confirmed the relations between the variables involved in CF and PR and supported the development of strategies to reduce nurses or nursing students’ CF, enhance professional quality of life, and, consequently, mitigate organizational turnover intention. All of the centrality scores are zero, which implied there was a lack of collaboration between authors. Also, the distribution of countries and institution maps can also reflect this issue (see Figure 4B).

### Author Analysis

**FIGURE 5:**
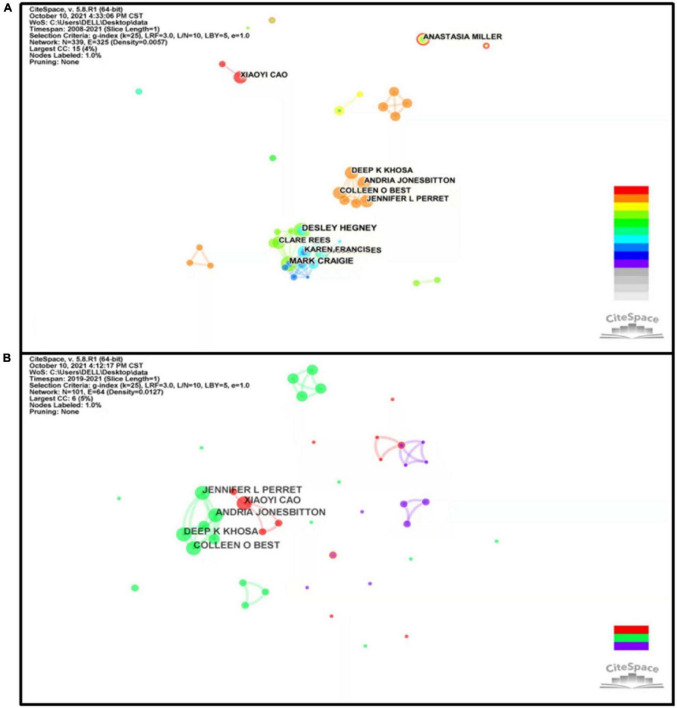
The network of co-authorship in the field of CF and PR during 2008–2021 **(A)** and the COVID-19 pandemic **(B)**. The individual circle indicates one author, and the size of the circle was weighted by the number of articles published by authors. The individual link between two circles indicates collaboration between two authors on the same article, and its thickness was weighted by the frequency of collaborations. The purple rims of circles indicate high betweenness centralities.

The map of authors was presented to reveal the most prolific authors and co-authors, and intuitively demonstrate the closeness of collaboration among the authors, which could provide information on influential research groups and potential collaborators and help researchers establish cooperative relationships. As described in Supplementary Table 4A, Craigie M (5 articles) published the most studies, followed by Hegney D (5 articles), Perret JL (4 articles), Rees C (4 articles), and Khosa DK (4 articles). Three of them are from Curtin University in Australia, and the rest are from the University of Guelph in Canada. Those implied authors publishing more articles are inclined to collaborate more closely with others, which is contrary to the results of the country and institution analysis. As one of the representative figures, Craigie Mand led his research team to investigate the influences of anxiety, stress, and depression and how they relate to compassion satisfaction and CF. Besides, they also evaluated the effectiveness of a psychosocial intervention designed to promote resilience among various occupational groups (nurses and rural general practitioners). In addition, we can see there are 339 nodes and 325 links in Figure 5A. A network density of only 0.0057 indicated the cooperation between authors in this field was not close enough. This issue can also be reflected by insignificant centrality scores (all of them are zero). If more authors can collaborate, more high-quality articles will be produced in the future. Thus, broader cooperation between authors and institutions should be encouraged to facilitate the development of the field of CF and PR.

During the COVID-19, a map of authors was generated including 101 nodes and 64 links (shown in Figure 5B and Supplementary Table 4B). There are approximately twice as many nodes as there are links in co-authorship, and it implies decreased cooperation. As shown in Supplementary Table 4B, only four authors entered the top 10. The top 5 authors were Khosa DK (4 articles), Perret JL (4 articles), Jonesbitton A (4 articles), Cao XY (4 articles), and Best CO (4 articles). Apart from Cao XY, the remaining authors all come from Canada. This was further evidenced that the researchers from Canada have devoted themselves to research in this area.

### Analysis of Journals

The analysis of journals will facilitate understanding the direction of specific research topics (Chuang and Ho, 2014). The Web of Science^®^ Core Collection showed that the 391 documents included covered 260 different journals over the past 14 years, all of which are widely distributed. We analyzed the top 15 active journals that published articles (Supplementary Table 5), accounting for 17.9% of 391 publications. The average impact factor (IF) of the top 15 journals in 2020 was 2.978. Frontiers in Psychology was the most productive journal (8 articles, 2020 IF = 2.990), followed by the British Journal of Social Work (7 articles, 2020IF = 1.884), and the International Journal of Environmental Research and Public Health (6 articles, 2020 IF = 3.390). Among them, eight of the top 15 journals specialize in social work and psychology, and four are nursing journals. From the analysis of journals, we inferred that studies in CF and PR fields mainly focused on nurses and social workers and their psychological problems, which can also be found from the analysis of categories. Nurses are usually to exposed various stressful work environments in daily work, such as the death of patients, violence, acute conditions, and the suffering of patients, which they are often unable to deal with (Yilmaz et al., 2018; Partlak Günüşen et al., 2021). For clinical social workers on the frontline, they are also faced with a serious human service crisis: clients with mental health problems exacerbated by the stress related to COVID-19 and individuals with no previous behavioral health issues suffering from symptoms due to social isolation, quarantine, and the drastic change in daily life. Therefore, they experience higher levels of CF symptoms than other service practitioners (Cavanagh et al., 2020; Felder, 2021).

**FIGURE 6:**
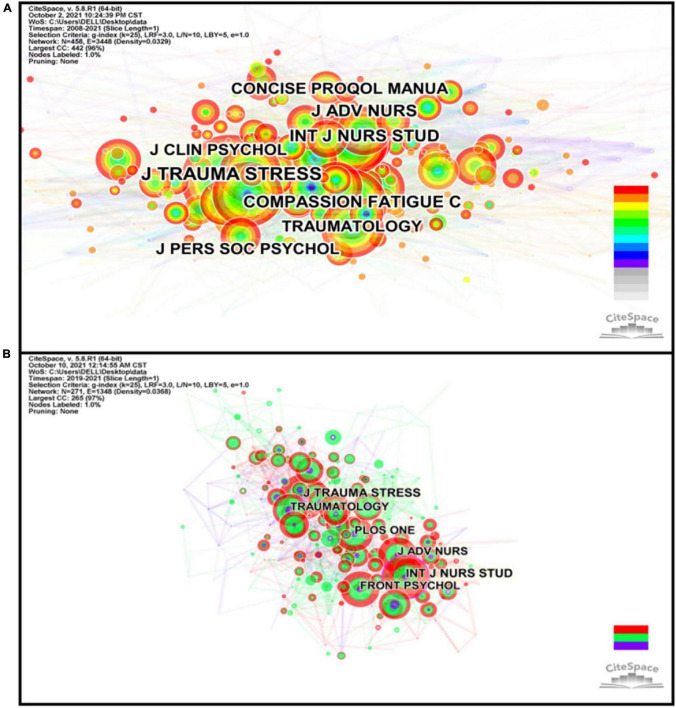
The network visualization map of journal co-citation analysis during 2008–2021 **(A)** and the COVID-19 pandemic **(B)**. The individual circle indicates a journal, and its size was weighted by the number of cited journals. The individual link between two circles meant co-citation relationships, and its thickness was weighted by the frequency of collaborations. The purple rims of circles indicate high betweenness centralities.

The map of cited journals was composed of 458 nodes and 3,448 links, including a total of 10,585 references (shown in Figure 6A and Supplementary Table 6A). The first in the frequency and centrality were Journal of Traumatic Stress and Anxiety Stress and Coping, respectively. In the journal cited in 167 records of Journal of Traumatic Stress, one literature review published by Dekel got the higher citation counts (Dekel and Goldblatt, 2008), which described the inter-generational transmission of post-traumatic stress disorder (PTSD) from fathers to sons in families of war veterans, and manifested fathers’ PTSD is a risk for increased emotional and behavioral problems among the children. Meanwhile, aiming to enhance the understanding of PTSD, the authors affirmed that future research should move from a description of the phenomenon to a better understanding of the factors that intensify or reduce it.

During the COVID-19, a cited journal map was generated by CiteSpace, which included 271 nodes and 1,348 links (shown in Figure 6B and Supplementary Table 6B). The first in the frequency and centrality were the Journal of Traumatic Stress and the International Journal of Nursing Studies, respectively. In the journal cited in 61 records of the Journal of Traumatic Stress, one review had the larger citation (Carmassi et al., 2020). This article summarized related risk and resilience factors for PTSD and post-traumatic stress symptoms in healthcare workers, aiming to constantly improve the effectiveness of intervention strategies.

### The Knowledge Base of a Research Field

#### Reference Co-citation Analyses

**FIGURE 7:**
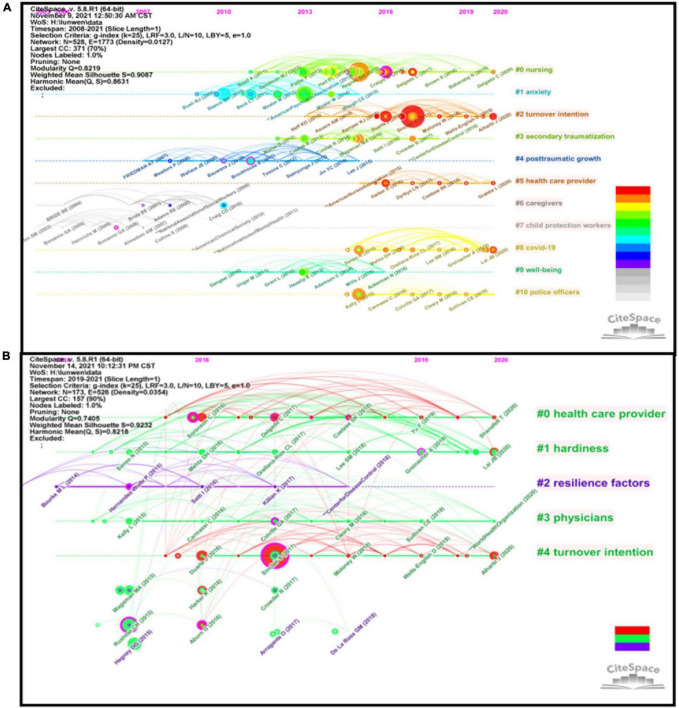
The timeline view of research focused on CF and PR during 2008–2021 **(A)** and the COVID-19 pandemic **(B)**. In the timeline view plot, keywords on the same horizontal line belong to the right cluster. The colors of lines and keywords are corresponding to the colors of the time slice at the top.

The knowledge base is composed of a collection of co-cited documents. A synthesized network of cited references and co-citation clusters of references was developed to reveal the intellectual base of a research area and the corresponding trend (Yuan et al., 2021). Table 1A documented 12 major clusters of reference co-citations (each cluster contains over 15 members), which are labeled by noun phrases from keywords of their citers of the cluster (Chen et al., 2010). The size was proportional to the number of members contained in a cluster, and more members indicated more representatives. Thus, clusters #0–#6 are very important owing to their larger sizes. As an indicator measuring the homogeneity of a cluster, the silhouette values ranged from 0.739 to 0.995, indicating highly homogeneous (Chen et al., 2010). A timeline visualization was also created to show the major 12 clusters along horizontal timelines, which helped us to form an overview of the evolution process of the CF and PR field over the years (Figure 7A). Clusters are numbered from 0, i.e., Cluster #0 is the largest cluster (nursing), and Cluster #10 is the smallest one (anxiety). For cited reference clusters, the map indicated that most clusters were concentrated from 2003 to 2020. The earlier research direction lay in clusters #7 (“child protection workers”) and #6 (“caregivers”), with most publications dated around 2006. This was followed by clusters #4 (“posttraumatic growth”), #1 (“anxiety”), and #9 (“well-being”), with most publications dated around 2000. Then, researchers shift their focus of attention to Clusters #0 (“nursing”) and #3 (“secondary traumatization”), with most publications dated around 2015. Clusters #10 (“police officers”), #8 (“COVID-19”), #5 (“health care”), and #2 (“turnover intention”) were the focus of the latest publications dated around 2017. Cluster #2 has a high concentration of nodes with citation bursts, which coincides with the fact that this is the latest formed cluster. Besides, Clusters #5 and #8 appear to have recent publications with citation bursts. Interestingly, we found that the sustainability of a specialty varies. For example, Cluster #0 sustains 11 years and remains active until now, whereas Clusters #0–1, 3–4, 6–7, 9, and 13 are relatively short-lived.

**TABLE 1:** Main clusters (>15 members) of reference co-citation analysis in the field of CF and PR.

(A) Main clusters (>15 members) of reference co-citation analysis in the field of CF and PR (from 2008 to 2021).						(B) Main clusters (>15 members) of reference co-citation analysis in the field of CF and PR (during the COVID-19 pandemic)					
Rank	Cluster ID	Size	Silhouette	Mean (year)	Theme	Rank	Cluster ID	Size	Silhouette	Mean (year)	Theme
1	0	66	0.739	2014	Nursing	1	0	31	0.835	2018	Health care provider
2	1	38	0.909	2011	Anxiety	2	1	21	0.995	2017	Hardiness
3	2	37	0.905	2017	Turnover intention	3	2	21	0.983	2015	Resilience factors
4	3	32	0.957	2015	Secondary traumatization	4	3	20	0.875	2016	Physicians
5	4	31	0.923	2011	Posttraumatic growth	5	4	20	0.956	2018	Turnover intention
6	5	31	0.894	2017	Health care provider						
7	6	26	0.977	2007	Caregivers						
8	7	25	0.99	2005	Child protection workers						
9	8	21	0.995	2017	COVID-19						
10	9	18	0.97	2013	Well-being						
11	10	17	0.95	2016	Police officers						

*WOS, Web of Science; CF, compassion fatigue; PR, psychological resilience.*

Human service practitioners such as nursing practitioners, physicians, therapists, child protection workers, police officers, and first responders often respond to potentially traumatic events or incidents and are requested to help traumatized individuals as part of their duties (Cluster #0, Cluster #5, Cluster #6, Cluster #7, and Cluster #10) (Musa and Hamid, 2008; Blau et al., 2012; Jacobson, 2012; Giada et al., 2017; Denkinger et al., 2018; Greinacher et al., 2019a; Kamijo et al., 2020; Maran et al., 2020; Vagni et al., 2020b; Smith et al., 2021). Recently, researchers have found that an individual may show positive changes following a traumatic event, such as treasuring life more, enhancement of close relationships, recognizing and elaborating on personal strengths, recognizing new possibilities, and spiritual development (Tedeschi and Calhoun, 1996; Tedeschi and Calhoun, 2004; Ogińska-Bulik and Kobylarczyk, 2015). This phenomenon is referred to as post-traumatic growth (PTG). Nevertheless, exposure to traumatized populations has severely tested their mental and physical health and compromised their well-being (Cluster #9) (Figley, 1995; Conrad and Kellar-Guenther, 2006; Bride et al., 2007; Carmassi et al., 2020). Among them, CF has traditionally been recognized as a common problem, awareness of this issue has been increased significantly since the COVID-19 emergency, which can also be partly reflected by the rapidly increased annual number of publications in the CF and PR field (Cluster #8) (Melvin, 2015; Burnett, 2017; Sinclair et al., 2017; Greinacher et al., 2019a; Ruiz-Fernández et al., 2020; Vagni et al., 2020a; Xie et al., 2021). CF is described as both STS and cumulative BO (Cluster #3)(Stamm, 2010). CF can leave caring professions cumulatively vulnerable to waning quality of care, difficult relationships with colleagues, or even cause more severe mental health symptoms, such as posttraumatic stress disorder, anxiety, or depression (Cluster #1)(Cocker and Joss, 2016; Iimura, 2022). Furthermore, several studies have shown that CF was significantly and positively correlated with turnover intention (Cluster #2) (Sung et al., 2012; Wells-English et al., 2019; Ådland et al., 2021; Cao and Chen, 2021a,b; Magnavita et al., 2021). One study conducted in China demonstrated that more than 60% of nursing practitioners manifested high to a significantly high level of turnover intention during the COVID-19 crisis. Thus, high turnover and poor retention become an important concern, especially for health care providers.

During the COVID-19 pandemic, the largest 5 clusters were discussed in our review (see Table 1B). The silhouette values ranged from 0.835 to 0.995, indicating that each cluster is highly homogeneous (Chen et al., 2010). As shown in the timeline overview (Figure 7B), the earlier research direction lay in clusters #2 (“resilience factors”) and #3 (“physicians”). This was followed by clusters #0 (“health care provider”), #1 (“hardiness”), and #4 (“turnover intention”). Except for clusters #2 and #5, the remaining is still active.

For health care providers, coping with the COVID-19 emergency can be psychologically stressing/is highly related to stress, and severe stress can accelerate the development of secondary trauma (Cluster 0 and Cluster 3) (Vagni et al., 2020a,b). PR is a protective factor preventing frontline healthcare professionals against secondary traumatization symptoms (Cluster 2) (Bartone, 2012; Jamal, 2017; Greinacher et al., 2019b; Ogińska-Bulik and Michalska, 2021). Also, it can weaken the negative impact of CF on health care workers’ job satisfaction and turnover intention (Cluster #4) (Alameddine et al., 2021; Antonsdottir et al., 2021; Labrague and de Los Santos, 2021). Furthermore, as a core component of PR, hardiness positively reframes negative experiences as opportunities and challenges to overcome and thus takes action to solve problems. This personality trait is beneficial in buffering exposure to extreme stress, thus, strategies that enhance hardness may be conducive to prompting PTG and well-being (Cluster #1) (Kobasa et al., 1982; Bartone and Bowles, 2021).

#### Most Cited Articles Analysis

The most cited references are usually viewed as landmarks based on their groundbreaking contributions. By analyzing these articles, the key knowledge foundation of each research field could be identified (Chen et al., 2019). Among the top 10 frequency of cited references, there are two books, two reviews, and six clinical studies. The majority of them (Flarity et al., 2013; Potter et al., 2013; Hart et al., 2014; Hunsaker et al., 2015; Kelly et al., 2015; Rushton et al., 2015; Sinclair et al., 2017) were published in Nursing Journal, indicating that the importance of these articles in this field, which also had a greater influence in this field. The most cited article in our dataset is Sinclair et al. (2017), which is also the more recent article on the list, suggesting that it has inspired intense interest in CF among healthcare providers (Sinclair et al., 2017). Additionally, two descriptive studies (Flarity et al., 2013; Potter et al., 2013), first examined the therapeutic effect of CF resiliency programs designed for oncology nurses and emergency nurses, respectively, and recommended nurses should develop resiliency skills that will enable them to manage CF effectively (see Table 2A).

**TABLE 2:** Main clusters (>15 members) of reference co-citation analysis in the field of CF and PR.

Rank	Author and publication year	Frequency	Title	Journal	IF (2020)	Quartile (2020)	Category
**(A) Top 10 highest cited articles in the field of CF and PR (during 2008–2021)**							
1	Sinclair et al., 2017	16	Compassion fatigue: a meta-narrative review of the healthcare literature	International Journal of Nursing Studies	5.837	Q1	Nursing
2	Rushton et al., 2015	15	Burnout and Resilience Among Nurses Practicing in High-Intensity Settings	American Journal of Critical Care	2.228	Q4	Nursing
3	Hegney et al., 2015	14	The contribution of individual psychological resilience in determining the professional quality of life of Australian nurses	Frontiers in Psychology	2.988	Q2	Psychology
4	American Psychiatric Association, 2013	13	Diagnostic and statistical manual of mental disorders	NA	NA	NA	NA
5	Hart et al., 2014	10	Resilience in nurses: an integrative review	Journal of Nursing Management	3.325	Q1	Nursing
6	Stamm, 2010	10	The Concise ProQOL Manual: the concise manual for the Professional Quality of Life Scale	NA	NA	NA	NA
7	Potter et al., 2013	9	Evaluation of a Compassion Fatigue Resiliency Program for Oncology Nurses	Oncology Nursing Forum	2.172	Q4	Nursing
8	Hunsaker et al., 2015	9	Factors That Influence the Development of Compassion Fatigue, Burnout, and Compassion Satisfaction in Emergency Department Nurses	Journal of Nursing Scholarship	3.176	Q1	Nursing
9	Kelly et al., 2015	9	Predictors of Compassion Fatigue and Compassion Satisfaction in Acute Care Nurses	Journal of Nursing Scholarship	3.176	Q1	Nursing
10	Flarity et al., 2013	8	The Effectiveness of an Educational Program on Preventing and Treating Compassion Fatigue in Emergency Nurses	Advanced Emergency Nursing Journal		Q4	Nursing
**(B) Top 10 highest cited articles in the field of CF and PR (during the COVID-19 pandemic)**							
1	Sinclair et al., 2017	16	Compassion fatigue: a meta-narrative review of the healthcare literature	International Journal of Nursing Studies	5.837	Q1	Nursing
2	Rushton et al., 2015	9	Burnout and Resilience Among Nurses Practicing in High-Intensity Settings	American Journal of Critical Care	2.228	Q4	Nursing
3	Hegney et al., 2015	9	The contribution of individual psychological resilience in determining the professional quality of life of Australian nurses	Frontiers in Psychology	2.988	Q2	Psychology
4	Duarte et al., 2016	8	Relationships between nurses’ empathy, self-compassion and dimensions of professional quality of life: a cross-sectional study	International Journal of Nursing Studies	5.837	Q1	Nursing
5	Wagaman MA	7	The Role of Empathy in Burnout, Compassion Satisfaction, and Secondary Traumatic Stress among Social Workers	Social Work	2.29	Q2	Social work
6	Kapoulitsas and Corcoran, 2015	6	Compassion fatigue and resilience: a qualitative analysis of social work practice	Qualitative Social Work	1.171	Q1	Social work
7	Alharbi et al., 2020b	6	Personal characteristics, coping strategies, and resilience impact on compassion fatigue in critical care nurses: A cross-sectional study	Nursing and Health Sciences	1.857	Q3	Nursing
8	Harker et al., 2016	6	Exploring resilience and mindfulness as preventative factors for psychological distress burnout and secondary traumatic stress among human service professionals	Work-a Journal of Prevention Assessment and Rehabilitation	1.505	Q4	Public, Environmental and Occupational Health
9	Lai et al., 2020	6	Factors Associated With Mental Health Outcomes Among Health Care Workers Exposed to Coronavirus Disease 2019	JAMA Network Open	8.485	Q1	Medicine, General and Internal
10	Ludick and Figley, 2016	6	Toward a Mechanism for Secondary Trauma Induction and Reduction: Reimagining a Theory of Secondary Traumatic Stress	Traumatology	NA	NA	NA

*WOS, Web of Science; CF, compassion fatigue; PR, psychological resilience.*

During the COVID-19, only 3 same publications entered the top 10 landmark articles (see Table 2B) (Hegney et al., 2015; Rushton et al., 2015; Sinclair et al., 2017). The majority of studies mainly focused on the understanding of the relationship among the variables involved in PR or/and CF (Alex et al., 2015; Hegney et al., 2015; Kapoulitsas and Corcoran, 2015; Rushton et al., 2015; Duarte et al., 2016; Harker et al., 2016; Alharbi et al., 2020a). Besides, Ludick and Figley (2016) explored the mechanism for secondary trauma induction and reduction. He and his team thoroughly described and justified the compassion fatigue resilience model, which provides the best estimate yet in depicting STS reactions. The model is likely to instruct trauma survivors and future trauma-exposed professionals to improve their secondary stress resilience and allows secondary stress management more effectively.

### Emerging Trends and Research Frontiers of Caregivers

#### Keyword Co-occurrence Analysis

**FIGURE 8:**
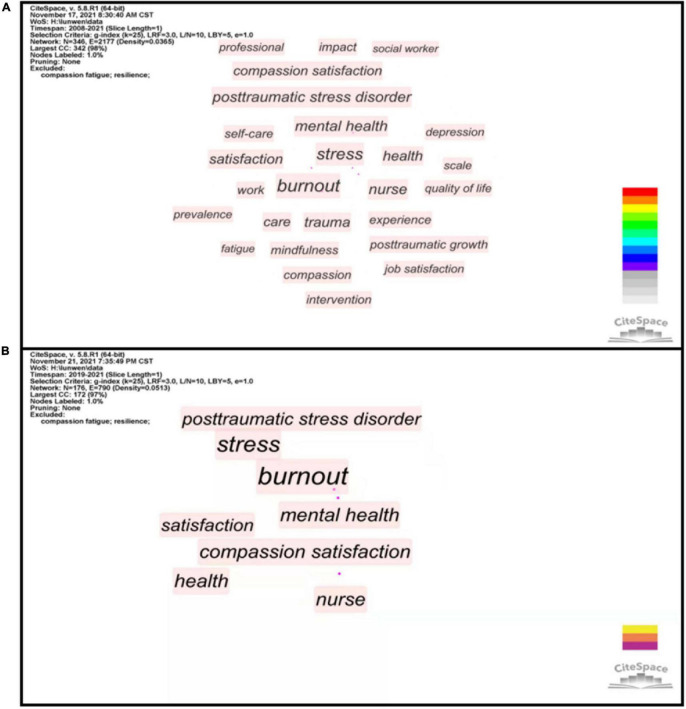
The co-occurrence network of keywords during 2008–2021 **(A)** and the COVID-19 pandemic **(B)**.

There were 348 co-occurrence keywords when we made the keyword co-occurrence analysis of data in set #4 (see Supplementary Appendix 1). We filtered out two conventional keywords including “compassion fatigue” and “resilience” and manually merged several synonyms, to make statistics clearer and more standardized. Keywords with low co-occurrence frequency did not relate to trending research topics, therefore, we set 20 as the threshold of co-occurrence frequency to filter out these keywords for a better analysis after an overall consideration. Figure 8A shows co-occurrence when thresholds were set to 20, including 26 keywords. This figure shows that nurses and social workers are the main target populations. Mental health problems (burnout, posttraumatic stress disorder, and depression) and personal or organizational effects of CF (job satisfaction and quality of life) became the research hotspots. At present, the survey is a common method to estimate the prevalence of CF and explored CF-related risk factors. The practice of self-care is an essential individual approach to building PR and increasing mindfulness, thus, diminishing CF and promoting personal growth (Beng et al., 2015; Kuglin Jones, 2017; Bonamer and Aquino-Russell, 2019; Blackburn et al., 2020).

During the COVID-19 pandemic (Figure 8B), there was little change in the distribution of co-occurrence keywords. This phenomenon implied the focus has not changed in the context of the epidemic.

### Strength and Limitation

There are several limitations in this study, which should be taken into consideration when interpreting the results. First, we only collected data from the Web of Science^®^ Core Collection when retrieving literature. Future studies should retrieve more databases to identify additional papers. Second, only articles and reviews were included in our analysis. Therefore, we have missed related articles in other document types. Third, bibliometrics is a quantitative analysis of academic publications, whereas articles with high citation counts do not necessarily equate to articles with high-quality or high-correlation with the target field. Researchers should use multi-method evaluations to obtain a more in-depth understanding of this research field. Fifth, only Citespace and traditional bibliometric indicators were used in the current study. Using a variety of research methods can make the results of the study more credible, and, therefore, more methods and indicators should be considered in the future.

## Conclusion

This was the first study to analyze the scientific output of research on the association of CF with PR using bibliometric analysis with CiteSpace^®^ software. Based on publications published from 2008 to 2021, we illustrated the change in this topic as the COVID-19 pandemic. Specifically, the number of publications in the CF and PR field presented an obviously ascending trend during the COVID-19 pandemic, indicating that CF should be especially emphasized for timely and effective delay and even prevent aggravation of psychological status through introducing some positive psychological sources such as PR. Overall, the United States was the most influential country in this research topic, as it not only published the largest number of articles but also had close cooperation with other countries, and therefore, more attention should be paid to enlarge the extrapolation of published findings. Moreover, more studies should also be performed in other countries and fields based on previously published studies. Curtin University in Australia was the most prolific institution, and Frontiers in Psychology was the most productive journal in this field. More researchers and practitioners should be trained under the supervision of specialists in this university, and more journals are also suggested to focus on this topic by launching a special issue. The turnover intention of health care providers has been a research focus in recent years. Nursing practitioners and social workers are currently the key target groups and will likely remain so in the near future. The mental problems of nursing practitioners and social workers are the main research topics. However, it is noted that this field is at an initial stage because limited research focusing on the association of CF with PR has been conducted, which is especially in developing countries. As an emerging research topic, scarce studies have focused on how to effectively prevent and relieve CF. Therefore, further studies and more collaborations among institutions and authors should be encouraged to explore effective interventions for the cultivation of PR and the reduction of CF.

## Data Availability Statement

The original contributions presented in the study are included in the article/Supplementary Material, further inquiries can be directed to the corresponding authors.

## Author Contributions

L-JY, YL, LT, XT, and MJ-H: conception and design. LC, XT, and MJ-H: administrative support. L-JY, YL, LT, and G-HW: collection and assembly of data. L-JY, G-HW, and S-WH: data analysis and interpretation. All authors wrote and approved the final manuscript.

## Author Disclaimer

The views expressed in this review come from the authors alone.

## Conflict of Interest

The authors declare that the research was conducted in the absence of any commercial or financial relationships that could be construed as a potential conflict of interest.

## Publisher’s Note

All claims expressed in this article are solely those of the authors and do not necessarily represent those of their affiliated organizations, or those of the publisher, the editors and the reviewers. Any product that may be evaluated in this article, or claim that may be made by its manufacturer, is not guaranteed or endorsed by the publisher.
