# Supplementary material for: A Bibliometric Analysis of the Association Between Compassion Fatigue and Psychological Resilience From 2008 to 2021
Source: Front Psychol. 2022 Jun 22;13:890327. doi: 10.3389/fpsyg.2022.890327 (PMC9258720; doi:10.3389/fpsyg.2022.890327)
Supplement: Supplementary file 1 [file Table_1.DOCX]

***Supplementary Material***

Contents of supplementary appendix

[Appendix 1 3](#_Toc97364879)

[Topic search queries used for data collection 3](#_Toc97364880)

[Appendix 2 5](#_Toc97364881)

[Top 10 Publications and Centrality of subject categories in the field of CF and PR 5](#_Toc97364882)

[Appendix 3 8](#_Toc97364883)

[Top 10 Publications and Centrality of Countries in the field of CF and PR 8](#_Toc97364884)

[Appendix 4 10](#_Toc97364885)

[Top 10 Publications of Institutions in the field of CF and PR 10](#_Toc97364886)

[Appendix 5 12](#_Toc97364887)

[Top 10 Prolific Authors in the field of CF and PR 12](#_Toc97364888)

[Appendix 6 14](#_Toc97364889)

[Top 15 Scholarly Journals in the field of CF and PR. 14](#_Toc97364890)

[Appendix 7 17](#_Toc97364891)

[Top 15 Frequency of Cited Journals in the field of CF and PR 17](#_Toc97364892)

##

## Appendix 1

## Topic search queries used for data collection

| **Web of Science Core Collection^®^ data base** | | |
| --- | --- | --- |
| # 1 | 3243 | TS= ("compassion fatigue") OR TS = ("vicarious traumas") OR TS = ("vicarious trauma") OR TS = ("secondary traumatic stress") OR TS = ("secondary traumatization") OR TS = ("secondary trauma") OR TS = ("secondary traumas") OR TS = ("secondary traumatizations") OR TS = ("vicarious traumatization")  Indexes = SCI-EXPANDED, SSCI, A&HCI, CPCI-S, ESCI, CPCI-SSH Timespan: 1900-2021 |
| # 2 | 112,473 | TS= (Resilience*)  Editions = A&HCI, ESCI, CPCI-SSH, CPCI-S, SCI-EXPANDED, SSCI Timespan: 1900-2021 |
| #3 | 414 | #1 AND #2  Editions = A&HCI, ESCI, CPCI-SSH, CPCI-S, SCI-EXPANDED, SSCI Timespan: 1900-2021 |
| # 4 | 391 | #1 AND #2 AND DOCUMENT TYPES: (Article OR Review)  Editions = A&HCI, ESCI, CPCI-SSH, CPCI-S, SCI-EXPANDED, SSCI Timespan: 1900-2021 |

##

## Appendix 2

## Top 10 Publications and Centrality of subject categories in the field of CF and PR

**Table S1. Top 10 Publications and Centrality of subject categories in the field of CF and PR.**

| **Table S1a.** Top 10 Publications and Centrality of subject categories in the field of CF and PR **(from 2008 to 2021)** | | | | | | **Table S1b.** Top 10 Publications and Centrality of subject categories in the field of CF and PR **(during the COVID-19 pandemic)** | | | | | |
| --- | --- | --- | --- | --- | --- | --- | --- | --- | --- | --- | --- |
| **rank** | **WOS categories** | **Frequency** | **rank** | **WOS categories** | **centrality** | **rank** | **WOS categories** | **Frequency** | **rank** | **WOS categories** | **centrality** |
| 1 | Psychology | 93 | 1 | Public, Environmental and Occupational Health | 0.36 | 1 | Nursing | 42 | 1 | Public, Environmental and Occupational Health | 0.38 |
| 2 | Nursing | 77 | 2 | Nursing | 0.27 | 2 | Psychology | 36 | 2 | Health Care Sciences and Services | 0.31 |
| 3 | Psychiatry | 58 | 3 | Psychology | 0.25 | 3 | Social Work | 22 | 3 | Nursing | 0.3 |
| 4 | Social Work | 48 | 4 | Psychiatry | 0.18 | 4 | Psychiatry | 17 | 4 | Education and Educational research | 0.16 |
| 5 | Psychology, Clinical | 45 | 5 | Medicine, General and Internal | 0.18 | 5 | Psychology, Clinical | 15 | 5 | Psychology | 0.15 |
| 6 | Psychology, Multidisciplinary | 30 | 6 | Social Work | 0.13 | 6 | Psychology, Multidisciplinary | 13 | 6 | Social Work | 0.1 |
| 7 | Public, Environmental and Occupational Health | 26 | 7 | Education and Educational research | 0.12 | 7 | Public, Environmental and Occupational Health | 13 | 7 | Social Sciences, Biomedical | 0.1 |
| 8 | Health Care Sciences and Services | 23（8） | 8 | Surgery | 0.11 | 8 | Education and Educational research | 11 | 8 | Biomedical Social Sciences | 0.1 |
| 9 | General and Internal Medicine | 23 | 9 | Health Care Sciences and Services | 0.08 | 9 | General and Internal Medicine | 11 | 9 | Veterinary Sciences | 0.09 |
| 10 | Family Studies | 21 | 10 | Veterinary Sciences | 0.07 | 10 | Family Studies | 11 | 10 | General and Internal Medicine | 0.08 |

*Abbreviations*: WOS = Web of science; CF = compassion fatigue; PR = psychological resilience.

## Appendix 3

## Top 10 Publications and Centrality of Countries in the field of CF and PR

**Table S2. Top 10 Publications and Centrality of Countries in the field of CF and PR.**

| **Table S2a.** Top 10 Publications and Centrality of Countries in the field of CF and PR **(from 2008 to 2021)** | | | | | | **Table S2b.** Top 10 Publications and Centrality of Countries in the field of CF and PR **(During the COVID-19 pandemic)** | | | | | |
| --- | --- | --- | --- | --- | --- | --- | --- | --- | --- | --- | --- |
| **rank** | **countries** | **Publications** | **rank** | **countries** | **centrality** | **rank** | **countries** | **Publications** | **rank** | **countries** | **centrality** |
| 1 | USA | 153 | 1 | USA | 0.41 | 1 | USA | 71 | 1 | USA | 0.63 |
| 2 | Australia | 61 | 2 | Canada | 0.36 | 2 | Canada | 19 | 2 | Canada | 0.45 |
| 3 | England | 36 | 3 | England | 0.22 | 3 | England | 19 | 3 | England | 0.35 |
| 4 | Canada | 31 | 4 | Australia | 0.17 | 4 | Australia | 16 | 4 | Germany | 0.2 |
| 5 | Israel | 25 | 5 | Germany | 0.16 | 5 | Italy | 11 | 5 | Israel | 0.17 |
| 6 | Italy | 13 | 6 | Spain | 0.12 | 6 | Israel | 8 | 6 | Spain | 0.16 |
| 7 | Peoples R China | 12 | 7 | Switzerland | 0.09 | 7 | Spain | 7 | 7 | Switzerland | 0.16 |
| 8 | South Korea | 11 | 8 | Netherlands | 0.08 | 8 | Turkey | 6 | 8 | Australia | 0.05 |
| 9 | Germany | 10 | 9 | Israel | 0.01 | 9 | Peoples R China | 6 | 9 | Italy | 0 |
| 10 | Spain | 10 | 10 | Ireland | 0.01 | 10 | Germany | 6 | 10 | Turkey | 0 |

*Abbreviations:* WOS = Web of science; CF = compassion fatigue; PR = psychological resilience.

## Appendix 4

## Top 10 Publications of Institutions in the field of CF and PR

**Table S3. Top 10 publications of institutions in the field of CF and PR.**

| **Table S3a.** Top 10 publications of institutions in the field of CF and PR **(from 2008 to 2021)** | | | **Table S3b.** Top 10 publications of institutions in the field of CF and PR **(During the COVID-19 pandemic)** | | |
| --- | --- | --- | --- | --- | --- |
| **Rank** | **Institutions** | **Publications** | **Rank** | **Institutions** | **Publications** |
| 1 | Curtin University | 14 | 1 | Arizona State University | 6 |
| 2 | Arizona State University | 6 | 2 | Tel Aviv University | 5 |
| 3 | Tel Aviv University | 6 | 3 | University of Guelph | 4 |
| 4 | Bar-Ilan University | 6 | 4 | University of Toronto | 4 |
| 5 | University of Sydney | 5 | 5 | Sichuan University | 4 |
| 6 | Ariel University | 5 | 6 | Canterbury Christ Church University | 3 |
| 7 | Guelph University | 4 | 7 | University of New England | 3 |
| 8 | Canterbury Christ Church University | 4 | 8 | Harvard Medical School | 3 |
| 9 | University of Toronto | 4 | 9 | Cent Queensland University | 3 |
| 10 | University of New England | 4 | 10 | Bar Ilan University | 3 |

Abbreviations: WOS = Web of science; CF = compassion fatigue; PR = psychological resilience.

##

## Appendix 5

## Top 10 Prolific Authors in the field of CF and PR

**Table 4. Top 10 Prolific Authors in the field of CF and PR.**

| **Table S4a.** Top 10 Prolific Authors in the field of CF and PR **(from 2008 to 2021)** | | | **Table S4b.** Top 10 Prolific Authors in the field of CF and PR **(During the COVID-19 pandemic)** | | |
| --- | --- | --- | --- | --- | --- |
| **rank** | **Author** | **Publications** | **rank** | **Author** | **Publications** |
| 1 | Mark Craigie | 5 | 1 | Deep K Khosa | 4 |
| 2 | Desley Hegney | 5 | 2 | Andria Jonesbitton | 4 |
| 3 | Jennifer L Perret | 4 | 3 | Jennifer L Perret | 4 |
| 4 | Clare Rees | 4 | 4 | Xiao Yi Cao | 4 |
| 5 | Deep K Khosa | 4 | 5 | Colleen O Best | 4 |
| 6 | Anastasia Miller | 4 | 6 | Valeria Giostra | 3 |
| 7 | Andria Jonesbitton | 4 | 7 | Jason B Coe | 3 |
| 8 | Karen Francis | 4 | 8 | Tiziana Maiorano | 3 |
| 9 | Clare S Rees | 4 | 9 | Amy L Greer | 3 |
| 10 | Xiao Yi Cao | 4 | 10 | Daniela Pajardi | 3 |

Abbreviations: WOS = Web of science; CF = compassion fatigue; PR = psychological resilience.

##

## Appendix 6

## Top 15 Scholarly Journals in the field of CF and PR (from 2008 to 2021).

**Table S5. Top 15 Scholarly Journals in the field of CF and PR (from 2008 to 2021).**

| **Rank** | **Journal** | **Record Count** | **IF (2020)** | **Quartile (2020)** |
| --- | --- | --- | --- | --- |
| 1 | Frontiers in Psychology | 8 | 2.990 | Q2 |
| 2 | British Journal of Social Work | 7 | 1.884 | Q2 |
| 3 | International Journal of Environmental Research and Public Health | 6 | 3.390 | Q2 |
| 4 | International Journal of Emergency Services | 5 | NA | Q2 |
| 5 | Child Abuse & Neglect | 4 | 3.928 | Q1 |
| 6 | Journal of Hospice & Palliative Nursing | 4 | 1.918 | Q2 |
| 7 | Journal of Interpersonal Violence | 4 | 6.144 | Q1 |
| 8 | Journal of Pediatric Oncology Nursing | 4 | 1.636 | Q3 |
| 9 | Sustainability | 4 | 3.251 | Q2 |
| 10 | American Journal of Hospice & Palliative Medicine | 4 | 2.500 | Q2 |
| 11 | American Journal of Orthopsychiatry | 4 | 2.364 | Q1 |
| 12 | Mindfulness | 4 | 4.684 | Q1 |
| 13 | Journal of Advanced Nursing | 4 | 3.187 | Q1 |
| 14 | Journal of Nursing Management | 4 | 3.325 | Q1 |
| 15 | Journal of Traumatic Stress | 4 | 3.476 | Q2 |

Abbreviations: WOS = Web of science; CF = compassion fatigue; PR = psychological resilience.

##

## Appendix 7

## Top 15 Frequency of Cited Journals in the field of CF and PR

**Table S6. Top 15 Frequency of Cited Journals in the field of CF and PR.**

| **Table S6a.** Top 15 Frequency of Cited Journals in the field of CF and PR **(from 2008 to 2021)** | | | | | | **Table S6b.** Top 15 Frequency of Cited Journals in the field of CF and PR **(During the COVID-19 pandemic)** | | | | | |
| --- | --- | --- | --- | --- | --- | --- | --- | --- | --- | --- | --- |
| **Rank** | **Cited Journals** | **Publications** | **Rank** | **Cited Journals** | **Centrality** | **Rank** | **Cited Journals** | **Publications** | **Rank** | **Cited Journals** | **Centrality** |
| 1 | Journal of Traumatic Stress | 167 | 1 | Anxiety, Stress, & Coping | 0.13 | 1 | Journal of Traumatic Stress | 61 | 1 | International Journal of Nursing Studies | 0.11 |
| 2 | Compassion Fatigue | 103 | 2 | Clinical Social Work Journa | 0.09 | 2 | International Journal of Nursing Studies | 59 | 2 | Clinical Psychology Review | 0.09 |
| 3 | International Journal of Nursing Studies | 98 | 3 | The American Psychologist | 0.09 | 3 | Frontiers in Psychology | 50 | 3 | Traumatology | 0.07 |
| 4 | Journal of Advanced Nursing | 91 | 4 | The American Journal of Psychiatry | 0.07 | 4 | Journal of Advanced Nursing | 48 | 4 | Journal of Clinical Psychology | 0.07 |
| 5 | Traumatology | 88 | 5 | Clinical Psychology Review | 0.06 | 5 | Plos ONE | 47 | 5 | International Journal of Environmental Research and Public Health | 0.07 |
| 6 | Journal of Clinical Psychology | 84 | 6 | Journal of the American Medical Association | 0.06 | 6 | Traumatology | 43 | 6 | Treating Compassion Fatigue | 0.07 |
| 7 | Journal of Personality and Social Psychology | 83 | 7 | International Journal of Nursing Studies | 0.05 | 7 | Journal of Clinical Psychology | 40 | 7 | Psychological Report | 0.07 |
| 8 | The Concise ProQOL Manual | 82 | 8 | Journal of Advanced Nursing | 0.05 | 8 | The Concise ProQOL Manual | 40 | 8 | Social Work | 0.06 |
| 9 | Professional Psychology-Research and Practice | 79 | 9 | Traumatology | 0.05 | 9 | Journal of Personality and Social Psychology | 38 | 9 | Personality and Individual Differences | 0.06 |
| 10 | Clinical Psychology Review | 71 | 10 | Journal of Clinical Psychology | 0.05 | 10 | Psychological Trauma-US | 38 | 10 | Child Abuse & Neglect | 0.06 |
| 11 | Plos ONE | 70 | 11 | Journal of Personality and Social Psychology | 0.05 | 11 | Journal of Nursing Management | 36 | 11 | International Journal of Stress Management | 0.05 |
| 12 | The American Journal of Orthopsychiatry | 69 | 12 | The American Journal of Orthopsychiatry | 0.05 | 12 | Journal of Clinical Nursing | 35 | 12 | Journal of Traumatic Stress | 0.05 |
| 13 | Compassion Fatigue | 35 | 13 | Psychological Trauma-US | 0.05 | 13 | Compassion Fatigue | 35 | 13 | Psychological Trauma-US | 0.05 |
| 14 | Anxiety, Stress, & Coping | 33 | 14 | Clinical Journal of Oncology Nursing | 0.05 | 14 | Anxiety, Stress, & Coping | 33 | 14 | Clinical Journal of Oncology Nursing | 0.05 |
| 15 | Depress Anxiety | 32 | 15 | Lancet | 0.05 | 15 | Depress Anxiety | 32 | 15 | Lancet | 0.05 |

Abbreviations: WOS = Web of science; CF = compassion fatigue; PR = psychological resilience.
